# Effects of working memory load and CS-US intervals on delay eyeblink conditioning

**DOI:** 10.1038/s41539-023-00167-w

**Published:** 2023-05-20

**Authors:** Leila Etemadi, Dan-Anders Jirenhed, Anders Rasmussen

**Affiliations:** 1 Neural Basis of Sensorimotor Control, Department of Experimental Medical Science, Lund, Sweden; 2 Associative Learning, Department of Experimental Medical Science, Lund, Sweden; 3 Erasmus Medical Center, Department of Neuroscience, Rotterdam, The Netherlands

**Keywords:** Classical conditioning, Human behaviour, Working memory

## Abstract

Eyeblink conditioning is used in many species to study motor learning and make inferences about cerebellar function. However, the discrepancies in performance between humans and other species combined with evidence that volition and awareness can modulate learning suggest that eyeblink conditioning is not merely a passive form of learning that relies on only the cerebellum. Here we explored two ways to reduce the influence of volition and awareness on eyeblink conditioning: (1) using a short interstimulus interval, and (2) having participants do working memory tasks during the conditioning. Our results show that participants trained with short interstimulus intervals (150 ms and 250 ms) produce very few conditioned responses after 100 trials. Participants trained with a longer interstimulus interval (500 ms) who simultaneously did working memory tasks produced fewer conditioned responses than participants who watched a movie during the training. Our results suggest that having participants perform working memory tasks during eyeblink conditioning can be a viable strategy for studying cerebellar learning that is absent of influences from awareness and volition. This could enhance the comparability of the results obtained in human studies with those in animal models.

## Introduction

Eyeblink conditioning is a widely used experimental paradigm for studying associative learning. In eyeblink conditioning, a subject learns to blink in response to a conditional stimulus (CS), such as a tone repeatedly followed by an unconditional blink eliciting stimulus (US). In delay eyeblink conditioning, the onset of the US starts before the end of the CS, whereas in trace eyeblink conditioning, there is a time gap between the end of the CS and the US onset. The timing of the conditional response (CR) is closely linked to the CS–US interval used during training, such that the closure of the eyelid occurs just before the expected US delivery, thus protecting the eye. Training with a short CS–US interval produces a learned blink response with a short latency, while a long CS–US interval will result in a CR with a longer latency^1,2^.

Lesioning and neurophysiological experiments show that in mammalian species such as rabbits, cats, ferrets, rats, and mice, the associative memory trace formed during delay eyeblink conditioning is located in the cerebellum^3–9^. Studies on cerebellar patients^10,11^ and neuroimaging experiments^12^ confirm that the cerebellum plays a pivotal role in eyeblink conditioning in humans as well. However, this does not mean that other brain parts are not involved in, or modulate, learning. In a previous study, we showed that participants, if prompted, can voluntarily produce what looks like conditioned blink responses even when there is no US^13^. But to what extent does this occur when participants are given no instructions? Trace conditioning seems to depend on the cerebellum but also the hippocampus and cerebrum^14,15^. This can explain why amnesic patients have deficits on trace but not delay conditioning^16,17^. However, another study showed that awareness of stimulus contingencies did not affect the learning rate on delay or trace conditioning^18^. Moreover, neuroimaging data indicate that extra-cerebellar brain regions are active during delay eyeblink conditioning^19^.

Noncerebellar involvement in humans could explain the discrepancy between learning curves in animals and humans. In humans, CRs often appear in the first ten trials, followed by only modest increases in responding^20–22^. Learning curves in animals, by contrast, exhibit a more gradual increase in the CR probability, and it is rare to see significant learning in the first few blocks^5,23,24^. In summary, there are still several unanswered questions about the neural circuit(s) responsible for eyeblink conditioning and the role of awareness, which remains a confounding variable. To relate cerebellar neurophysiological mechanisms to eyeblink conditioning in animal and human participants, it is desirable to reduce the influence of awareness and volition as much as possible.

Here we tested two strategies to reduce the influence of awareness and volition on conditioning in human participants. The first strategy was to use a short CS–US interval to minimize the time for participants to respond voluntarily. The second strategy was to reduce cortical contributions to the learning by presenting concurrent working memory tasks during conditioning. We chose working memory tasks that previous research suggests occupy the cortex but not the cerebellum.

## Results

### Linear mixed effects model

To test the effects of training, the interstimulus interval (ISI), and working memory tasks on the percentage of conditioned responses, we modeled CR percentage using a linear mixed effects model. A linear mixed effects model was used rather than a repeated measures analysis of variance because it is statistically more robust, considers individual differences, and copes with missing data points^25,26^. As fixed effects, we used the training block (1–10), ISI (150, 250, or 500 ms), sex (male or female), and whether or not the subject did working memory tasks (yes or no). We also included subject ID as a random effect in the model. The model was built in MATLAB (Mathworks Inc.) using the fitlme function with the formula: CRs ~1 + Block + Sex + WM + ISI + (1 | Subject).

**Fig. 1:**
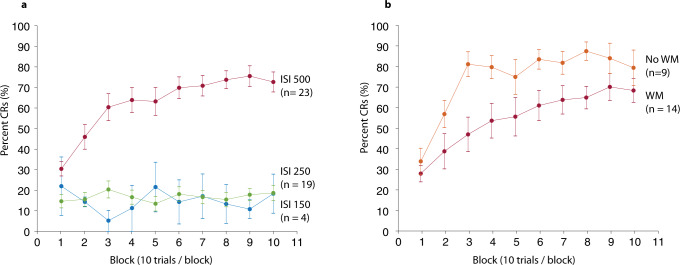
Effect of training, ISI, and working memory tasks on the percentage of CRs. **a** Displays the percentage of CRs (mean ± SEM) over 10 blocks of training for participants trained with a CS–US interval of 150 ms (yellow), 250 ms (red), and 500 ms (blue). **b** Displays the percentage of CRs (mean ± SEM) over 10 blocks of training for participants trained with a CS–US of 500 ms who either did (red) or did not (blue) perform working memory tasks during the conditioning.

The model shows that block, ISI, and WM tasks have a significant effect on the percentage of conditioned responses. For each block of 10 trials, the CR percentage increases by an average of 2.15%. Over ten blocks, this translates to 2.15*10 = 21.5%. This change is statistically significant (*t* = 8.4339, *P* = 4.4686e–16***, CI = 1.65–2.65%). Our model also showed that the interstimulus interval affects the CR percentage (Fig. 1a). A 1 ms increase in the ISI results in a 0.17% increase in the percentage of CRs. Switching from a 150 ms ISI to a 500 ms ISI translates to a 0.17*350 = 59.5% increase in CR percentage. This effect is also significant (*t* = 10.30, *P* = 1.74e–22***, CI = 0.13–0.20%). However, contrary to our previous findings^20^, sex did not affect CRs (*t* = 1.621, *P* = 0.11, CI = −1.67 to 17.4%).

### Awareness and working memory

After the training session, participants in the working memory group were asked, “have you noticed any relation between air puffs and tone?” Of the 11 subjects in this group, 3 said they had noticed that sometimes the stimuli came together and sometimes alone, but nothing more. Six of the 11 participants thought the stimuli were annoying and distracting. None of the 11 participants reported anything about the temporal relation between the stimuli. Despite this, conditioned responses were successfully acquired. Explicit awareness was thus not necessary for successful conditioning. Whether or not the participants solved working memory tasks during the conditioning affected the CR percentage (illustrated in Fig. 1b). Those who performed working memory tasks produced 13.68% fewer CRs than those who watched a film. This difference was statistically significant (*t* = 2.77, *P* = 0.0057**, CI = 3.99–23.4%). In a separate model, which included only participants that did the working memory tasks, we tested whether a subject’s relative ranking on working memory performance predicted the percentage of CRs. The results showed this was not the case (*t* = 1.3189, *P* = 0.18, CI = −0.9 to 5.0%).

### Summary

In summary, the results show that: (1) training results in more CRs; (2) a longer CS–US interval results in more CRs; (3) sex does not affect CR percentage; (4) doing a working memory task reduces the percentage of CRs; and (5) how well participants perform on the working memory tasks do not affect the CR percentage.

## Discussion

The two main findings of this study are that (1) an interstimulus interval <250 ms produces very few conditioned responses, and (2) having participants do working memory tasks reduces the learning rate. The lack of learning with a short CS–US interval goes against results in animal models where CS–US intervals between 150 ms and 500 ms typically produce high rates (80–100%) of conditioned responses^23,24,27–29^. However, it is largely consistent with previous research on humans where interstimulus intervals of <250 ms are associated with poor learning^1,21^, although in one study, 200 ms did produce some learning^30^. Given that we only tested four participants with a 150 ms ISI, we are not able to rule out that learning can occur with such a short ISI. However, the lack of learning to both 150 ms and 250 ms ISIs, combined with results from previous studies, indicate that using short CS–US intervals does not support eyeblink conditioning in humans. Furthermore, this also means that short using short ISIs is not a viable strategy to reduce active influences on eyeblink conditioning because no conditioning occurs, at least not when the training consists of ~100 trials.

Training with a 500 ms ISI did induce learning. In the control group (without the working memory distraction), there was a rapid increase in rates of responding. Within the first three blocks, the response rate reached an average of >80%, which did not change much for the remaining seven blocks. This is in line with other observations in eyeblink conditioning with human participants^11,19,20,22,30–32^. However, it differs from conditioning patterns in other mammals. In intact rabbits, rats, and mice, conditioned responses are sometimes seen only after several days of training^5,24,27,28,33^. Peak rates of responding and plateauing of the average learning curve usually take additional training days. It is conceivable that training humans for a longer time or on multiple occasions—as is traditionally done in animals—may result in higher rates of conditioned responses.

Doing working memory tasks during training with the 500 ms CS–US interval rendered participants unaware of the stimulus contingencies but did not prevent learning. Since we did not ask participants who did not do working memory tasks about their awareness, we cannot say if they were aware of the stimulus contingencies. However, our experience is that participants quickly realize that the airpuff comes after the sound, which is also consistent with observations in ref. ^18^. The learning curve of participants in the working memory group reached a plateau later than in the control group. The gradual increase in conditioned responses extended over nine of the ten blocks of training. Also, though learning was more gradual and the rates of responding were lower from the first block, the response rate eventually reached the same level ( > 80%) as the control group by the end of the ten blocks of training. Our results contradict earlier experiments where a masking task did not affect the learning rate^18^. However, in the earlier study, participants were asked to repeat words. Our working memory tasks were likely significantly more difficult.

In summary, previous experiments show that awareness of the stimulus conditions and voluntary blinking may be a source of accelerated learning during delay eyeblink conditioning. This study suggests that having participants do concurrent working memory tasks during conditioning may reduce active influences on learning, which may yield a learning process that is more purely cerebellar-dependent and thus more comparable to conditioned blink responses reported in the animal literature.

## Methods

### Participants

The participants were 42 students (females = 22; males = 20) at Lund University. The age range was 24.6 ± 5.38 years (mean ± SD). The participants were divided into four distinct groups (see Table 1). Three groups were trained with three different CS–US intervals: 150, 250, and 500 ms. The fourth group was trained with a 500 ms interval, but in addition to conditioning, participants were instructed to perform working memory (WM) tasks while being trained. Before the experiment, the subject signed a written consent form stating that they had been informed about the procedure in general terms, i.e., that their blink responses would be recorded and that they would be presented with tones and air puffs aimed at the eye. The consent form also verified that the subject knew that they could withdraw their participation at any time. As a token of gratitude, the subject received a cinema ticket at the end of the experiment (regardless of whether they had completed the entire protocol). The local ethical committee (Regionala etikprövningsnämnden Lund) approved the study (dnr 2017–785).

**Table 1:** Participants (total = 42) in each CS–US interval group and the group performing working memory (WM) tasks concurrently.

	Experimental groups, *n* = 33			Control group, *n* = 9
ISI	150 ms	250 ms	500 ms	500 ms
Condition	Movie	Movie	Working memory	Movie
Sample size	*n* = 4	*N* = 18	*n* = 11	*N* = 9

### Materials

**Fig. 2:**
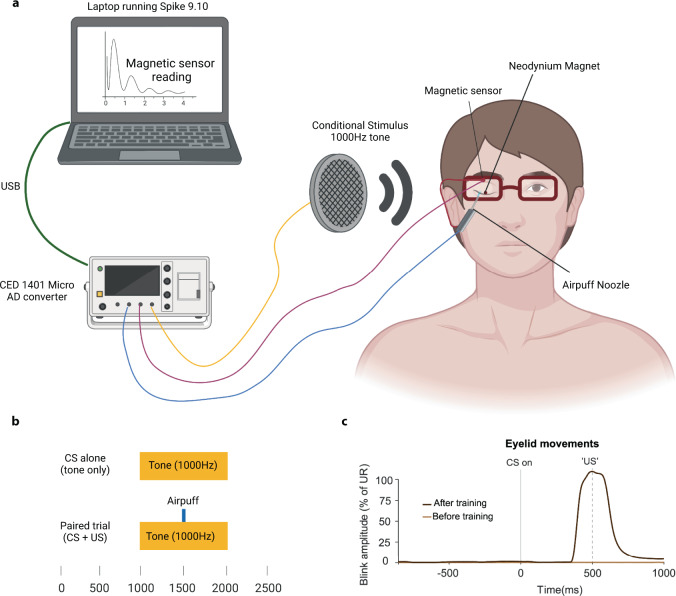
Setup, protocol, and example traces. **a** A Micro 1401 (CED) AD converter was used to trigger the tone from the speaker, the delivery of the airpuff, and sample data from the magnetic sensor. The sound was played from a portable loudspeaker. The pressure of the airpuff was delivered through a nozzle attached to a pair of glasses worn by the participant. **b** The training protocol consisted of 80% paired trials and 20% CS alone (probe) trials. **c** Example of smoothed raw data traces on CS alone trials before and after training.

The experimental setup is illustrated in Fig. 2. To detect eyelid movements, a small round neodymium magnet (diameter: 3 mm; thickness: 1 mm) was attached to the subject’s left eyelid using double stick tape. The resulting changes in the magnetic field were recorded using a GMR chip (AAH002-02E, NVE Corporation). The GMR chip and the nozzle delivering the airpuff were attached to the right side of a pair of glasses the subject wore during the test. The GMR sensor data were sampled at 1000 Hz and transferred to the computer via a Micro 1401 AD converter (Cambridge Electronic Design). The Micro 1401 was also used to trigger the loudspeakers playing the tone—a 1000 Hz tone lasting 1 s—and the opening of the D132202 solenoid valve (Aircom), releasing the airpuff.

### Eyeblink conditioning

The experiments were conducted in a quiet room on campus. For each subject, we adjusted the intensity of the airpuff so that it reliably elicited a reflexive blink response without causing irritation of the eye. The resulting pressure ranged from 0.5 to 1 bar. Likewise, the volume of the tone was adjusted to be audible but not unpleasant. Each individual received a total number of 100 trials (10 blocks of 10 trials). Of these 100 trials, 25% were probe trials meaning that the CS was presented alone. The intertrial interval was 10 ± 2 s.

In the groups that did not perform working memory tasks, participants were asked to choose a favorite TV show to watch on a laptop during the conditioning session. The subject was asked to concentrate on the program and to try not to control eyelid movements. In the group that received working memory tasks during conditioning, participants were told to focus and perform as well as possible on the working memory tasks. They were told that the purpose of the experiment was to see the effects on the working memory performance of distracting stimuli in the form of tones and air puffs. The goal was to have the subject perceive the experiment to be a test of working memory and to be unaware that it was, in fact, an eyeblink conditioning experiment. After the training, participants in the working memory group were asked if they had noticed any pattern in the presentation of the stimuli.

### Working memory tasks

Participants in the working memory group were given demanding working memory tasks during the eyeblink conditioning session. We selected some of the most common working memory tests that were possible to combine with the conditioning protocol, and that did not directly require timing and motor skills. Nevertheless, there is evidence showing that several of these working memory tasks do involve the cerebellum^34,35^. Specifically, participants did the following eight tests in the following order: (1) Corsi, (2) mental rotation, (3) multitask, (4) n-back (3-back), (5) Navon, (6) Stroop, (7) visual search, and the (8) Wisconsin card sorting task. The working memory tests were run on PsyToolkit online software (PsyToolkit is developed by and belongs to Professor Gijsbert Stoet and is available at www.psytoolkit.org). Each working memory task started with on-screen instructions provided by PsyToolkit. Complementary information was presented verbally when requested by participants. Each test started with a few training examples (provided by the software), and after that, the testing started. Responses were made using a computer keyboard and a mouse. When a participant had completed all 8 tests, they were instructed to start over with the first test. Participants usually completed 1.5 rounds of the tests. Results from the second round were not analyzed further.

### Data analysis

Eyeblink data was collected using the Spike2 v9.10 software (CED). The data from the working memory tasks were saved in Microsoft Excel. All data were subsequently exported to and analyzed in Matlab R2022a (Mathworks). Using custom Matlab scripts, we categorized each trial as (1) CR, (2) no CR, or (3) invalid trial. If a CR was present, the script estimated the onset and the peak of the response. All sweeps were checked manually to ensure that the script had made the correct categorizations. Errors were corrected manually. For the analysis of working memory test performance, we chose two variables: reaction time (RT) and success in the test (correct answers in %). The rank was computed using the rank function from Microsoft Excel software, and an average rank presented one value for each variable of RT and success in the test per person.

### Reporting summary

Further information on research design is available in the Nature Portfolio Reporting Summary linked to this article.

## Supplementary information

Reporting summary checklist

## Data Availability

Our GitHub data repository includes data from the experiments. The GitHub repository can be accessed at: https://github.com/rasmussenanders/ISI-WM/.

## Code availability

Our GitHub data repository includes the Matlab code used to analyze the data and generate the figures. The GitHub repository can be accessed at: https://github.com/rasmussenanders/ISI-WM/.
