# Supplementary material for: Effects of working memory load and CS-US intervals on delay eyeblink conditioning
Source: NPJ Sci Learn. 2023 May 20;8:16. doi: 10.1038/s41539-023-00167-w (PMC10199915; doi:10.1038/s41539-023-00167-w)
Supplement: Supplementary file 1 — Reporting summary checklist [file 41539_2023_167_MOESM1_ESM.pdf]

nature portfolio

Corresponding author(s): Anders Rasmussen

Last updated by author(s): Apr 6, 2023

Reporting Summary

Nature Portfolio wishes to improve the reproducibility of the work that we publish. This form provides structure for consistency and transparency in reporting. For further information on Nature Portfolio policies, see our Editorial Policies and the Editorial Policy Checklist.

Statistics

For all statistical analyses, confirm that the following items are present in the figure legend, table legend, main text, or Methods section.

n/a	Confirmed	
☐	☒	The exact sample size (*n*) for each experimental group/condition, given as a discrete number and unit of measurement
☐	☒	A statement on whether measurements were taken from distinct samples or whether the same sample was measured repeatedly
☐	☒	The statistical test(s) used AND whether they are one- or two-sided<br>*Only common tests should be described solely by name; describe more complex techniques in the Methods section.*
☐	☒	A description of all covariates tested
☐	☒	A description of any assumptions or corrections, such as tests of normality and adjustment for multiple comparisons
☐	☒	A full description of the statistical parameters including central tendency (e.g. means) or other basic estimates (e.g. regression coefficient) AND variation (e.g. standard deviation) or associated estimates of uncertainty (e.g. confidence intervals)
☐	☒	For null hypothesis testing, the test statistic (e.g. *F*, *t*, *r*) with confidence intervals, effect sizes, degrees of freedom and *P* value noted<br>*Give P values as exact values whenever suitable.*
☒	☐	For Bayesian analysis, information on the choice of priors and Markov chain Monte Carlo settings
☐	☒	For hierarchical and complex designs, identification of the appropriate level for tests and full reporting of outcomes
☐	☒	Estimates of effect sizes (e.g. Cohen's *d*, Pearson's *r*), indicating how they were calculated

Our web collection on statistics for biologists contains articles on many of the points above.

Software and code

Policy information about availability of computer code

Data collection	Spike2 v9.10 (Cambridge Electronics Design)
Data analysis	Spike2 v9.10 (Cambridge Electronics Design); Matlab 2022a (Mathworks)

For manuscripts utilizing custom algorithms or software that are central to the research but not yet described in published literature, software must be made available to editors and reviewers. We strongly encourage code deposition in a community repository (e.g. GitHub). See the Nature Portfolio guidelines for submitting code & software for further information.

Data

Policy information about availability of data

All manuscripts must include a data availability statement. This statement should provide the following information, where applicable:

- Accession codes, unique identifiers, or web links for publicly available datasets
- A description of any restrictions on data availability
- For clinical datasets or third party data, please ensure that the statement adheres to our policy

Data and code has been uploaded to github (https://github.com/rasmussenanders/ISI-WM). If accepted this repostitory will be made public

Human research participants

Policy information about studies involving human research participants and Sex and Gender in Research.

Reporting on sex and gender	We have consistently used the term sex ("kön" in Swedish). In the paper we did not find any sex differences which we report. All participants consented to all the analyses performed
Population characteristics	The participants were 42 students (females=22; males=20) at Lund University. The age range was 24.6 ± 5.38 years (mean ± SD).
Recruitment	Participants were university students
Ethics oversight	Regionala etikprövningsnämnden Lund approved the study (dnr 2017-785).

Note that full information on the approval of the study protocol must also be provided in the manuscript.

Field-specific reporting

Please select the one below that is the best fit for your research. If you are not sure, read the appropriate sections before making your selection.

☐ Life sciences ☒ Behavioural & social sciences ☐ Ecological, evolutionary & environmental sciences

For a reference copy of the document with all sections, see nature.com/documents/nr-reporting-summary-flat.pdf

Behavioural & social sciences study design

All studies must disclose on these points even when the disclosure is negative.

Study description	A combination of survey data and quantitative data
Research sample	Participants were Lund University students. Since we were investigating fundamental motor learning phenomena, students are fulfill all requirements
Sampling strategy	Convenience sampling. Rough statistical power estimates were based on previous experiments combined with assessment of what effect sizes would be interesting.
Data collection	Materials<br>The experimental setup is illustrated in Figure 1. To detect eyelid movements, a small round neodymium magnet (diameter: 3 mm; thickness: 1 mm) was attached to the subject's left eyelid using double stick tape. The resulting changes in the magnetic field were recorded using a GMR chip (AAH002-02E, NVE Corporation). The GMR chip and the nozzle delivering the air puff were attached to the right side of a pair of glasses the subject wore during the test. The GMR sensor data was sampled at 1000Hz and transferred to the computer via a Micro1401 AD converter (Cambridge Electronic Design). The Micro 1401 was also used to trigger the loudspeakers playing the tone – a 1000 Hz tone lasting 1 second – and the opening of the D132202 solenoid valve (Aircom), releasing the air puff.<br><br>Eyeblink conditioning<br>The experiments were conducted in a quiet room on campus. For each subject, we adjusted the intensity of the air puff so that it reliably elicited a reflexive blink response without causing irritation of the eye. The resulting pressure ranged from 0.5 to 1 bar. Likewise, the volume of the tone was adjusted to be audible but not unpleasant. Each individual received a total number of 100 trials (10 blocks of 10 trials). Of these 100 trials, 25% were probe trials meaning that the CS was presented alone. The intertrial interval was 10±2 seconds.<br><br>In the groups that did not perform working memory tasks, participants were asked to choose a favorite TV show to watch on a laptop during the conditioning session. The subject was asked to concentrate on the program and to try not to control eyelid movements. In the group that received working memory tasks during conditioning, participants were told to focus and perform as well as possible on the working memory tasks. They were told that the purpose of the experiment was to see the effects on working memory performance of distracting stimuli in the form of tones and air puffs. The goal was to have the subject perceive the experiment to be a test of working memory and to be unaware that it was, in fact, an eyeblink conditioning experiment. After the training, participants in the working memory group were asked if they had noticed any pattern in the presentation of the stimuli.<br><br>Data analysis<br>Eyeblink data was collected using the Spike2 v9.10 software (CED). The data from the working memory tasks were saved in Microsoft Excel. All data were subsequently exported to and analyzed in Matlab R2022a (Mathworks). Using custom Matlab scripts, we categorized each trial as (1) CR, (2) no CR, or (3) invalid trial. If a CR was present, the script estimated the onset and the peak of the response. All sweeps were checked manually to ensure that the script had made the correct categorizations. Errors were corrected manually. For the analysis of working memory test performance, we chose two variables: reaction time (RT) and success in the test (correct answers in %). The rank was computed using the rank function from Microsoft Excel software, and an average rank

	presented one value for each variable of RT and success in the test per person.
Timing	October 2018 - February 2019 – No major gap
Data exclusions	No participants were excluded
Non-participation	No participants dropped out
Randomization	We tested the different ISIs in order – first 150ms, then 250ms, then 500ms. The demographics in the different experimental groups is similar

Reporting for specific materials, systems and methods

We require information from authors about some types of materials, experimental systems and methods used in many studies. Here, indicate whether each material, system or method listed is relevant to your study. If you are not sure if a list item applies to your research, read the appropriate section before selecting a response.

Materials & experimental systems

n/a	Involved in the study
☒	☐ Antibodies
☒	☐ Eukaryotic cell lines
☒	☐ Palaeontology and archaeology
☒	☐ Animals and other organisms
☒	☐ Clinical data
☒	☐ Dual use research of concern

Methods

n/a	Involved in the study
☒	☐ ChIP-seq
☒	☐ Flow cytometry
☒	☐ MRI-based neuroimaging
